# Could Curdlan/Whey Protein Isolate/Hydroxyapatite Biomaterials Be Considered as Promising Bone Scaffolds?—Fabrication, Characterization, and Evaluation of Cytocompatibility towards Osteoblast Cells In Vitro

**DOI:** 10.3390/cells11203251

**Published:** 2022-10-16

**Authors:** Katarzyna Klimek, Krzysztof Palka, Wieslaw Truszkiewicz, Timothy E. L. Douglas, Aleksandra Nurzynska, Grazyna Ginalska

**Affiliations:** 1 Chair and Department of Biochemistry and Biotechnology, Medical University of Lublin, Chodzki 1 Street, 20-093 Lublin, Poland; 2 Faculty of Mechanical Engineering, Lublin University of Technology, Nadbystrzycka 26 Street, 20-618 Lublin, Poland; 3 School of Engineering, Lancaster University, Gillow Avenue, Lancaster LA1 4YW, UK; 4 Materials Science Institute (MSI), Lancaster University, Lancaster LA1 4YW, UK

**Keywords:** bone tissue engineering, bone scaffolds, bone defects, curdlan, whey protein isolate, bioactive materials, cytocompatibility, cytotoxicity, osteoblast proliferation, osteoblast differentiation

## Abstract

The number of bone fractures and cracks requiring surgical interventions increases every year; hence, there is a huge need to develop new potential bone scaffolds for bone regeneration. The goal of this study was to gain knowledge about the basic properties of novel curdlan/whey protein isolate/hydroxyapatite biomaterials in the context of their use in bone tissue engineering. The purpose of this research was also to determine whether the concentration of whey protein isolate in scaffolds has an influence on their properties. Thus, two biomaterials differing in the concentration of whey protein isolate (i.e., 25 wt.% and 35 wt.%; hereafter called Cur_WPI25_HAp and Cur_WPI35_HAp, respectively) were fabricated and subjected to evaluation of porosity, mechanical properties, swelling ability, protein release capacity, enzymatic biodegradability, bioactivity, and cytocompatibility towards osteoblasts in vitro. It was found that both biomaterials fulfilled a number of requirements for bone scaffolds, as they demonstrated limited swelling and the ability to undergo controllable enzymatic biodegradation, to form apatite layers on their surfaces and to support the viability, growth, proliferation, and differentiation of osteoblasts. On the other hand, the biomaterials were characterized by low open porosity, which may hinder the penetration of cells though their structure. Moreover, they had low mechanical properties compared to natural bone, which limits their use to filling of bone defects in non-load bearing implantation areas, e.g., in the craniofacial area, but then they will be additionally supported by application of mechanically strong materials such as titanium plates. Thus, this preliminary in vitro research indicates that biomaterials composed of curdlan, whey protein isolate, and hydroxyapatite seem promising for bone tissue engineering applications, but their porosity and mechanical properties should be improved. This will be the subject of our further work.

## 1. Introduction

Bone, a hard tissue forming the body’s skeleton, is often prone to damage and fractures, due to increasing age of patients, traumas, accidents, intensive and improperly performed physical activity, or diseases. Globally, it is considered to be the second most commonly transplanted tissue. Conventional strategies for the replacement of damaged bone tissue include the use of auto-, allo-, and xenografts [1,2,3,4,5]. In spite of many favorable features of these techniques, they also possess many drawbacks, which highly restrict their use. For this reason, bone tissue engineering (BTE)—a modern therapeutical approach—is constantly being developed. Every year various bone scaffolds are designed in order to achieve products which will meet the requirements for biomaterials intended for BTE applications [6,7,8,9].

The fundamental feature of bone scaffolds is biocompatibility, i.e., compatibility with biological fluids, cells, and tissues. Thus, a biocompatible biomaterial cannot be overly cytotoxic (locally and systemically), genotoxic, immunogenic, mutagenic, cancerogenic or thrombogenic. Moreover, a biocompatible biomaterial should have osteoinductive and osteoconductive properties as well as undergoing osseointegration with surrounding bone tissue [10,11,12,13,14]. Osteoinductivity is defined as the biomaterial’s ability to promote new bone formation via molecular and mechanical stimuli as well as its capacity to recruit osteoprogenitor cells and promote their differentiation towards osteogenic cells. Osteoconductivity is determined as a biomaterial’s ability to promote adhesion and proliferation of osteoblasts as well as support creation of extracellular matrix (ECM) on its surface by these cells. In turn, a bioactive biomaterial has the ability to form an apatite layer on its surface, which enables a tight connection between scaffold and bone (i.e., osseointegration) [15,16,17,18]. The aforementioned properties strictly depend on a biomaterial’s composition and its structural, physicochemical as well as mechanical properties [19].

Within this research, two biomaterials composed of curdlan, whey protein isolate (WPI), and hydroxyapatite (HAp) granules were fabricated using a technically straightforward thermal method. The biomaterials differed from each other by the content of WPI, namely one of them contained 25 wt.% WPI, while the second contained 35 wt.% WPI. Then, structural, mechanical as well as biological characterization was performed in order to evaluate the influence of WPI content on properties of the scaffolds, and as a consequence, on their potential as scaffolds for BTE applications. Curdlan (β-1,3-glucan) is a non-toxic, natural polysaccharide, increasingly used in tissue engineering [20,21,22,23]. WPI is a waste product in the dairy industry, as it is obtained during cheese manufacturing. It is rich in proteins, especially in β-lactoglobulin [24,25]. To date, there are a few papers which describe the application of WPI in BTE [24,25,26,27,28]. In turn, synthetic HAp granules are most often used as a component of bone scaffolds, thanks to their similarities with natural HAp occurring in bones [21]. It is worth noting that the choice of the ingredients of the presented scaffolds was motivated by our research experiences. In our previous paper, we characterized bone scaffolds composed of curdlan and HAp granules. It was found that the cytocompatibility of a thermally obtained curdlan/HAp biomaterial (marked as glu/HA T) is limited, because the osteoblasts only grew on HAp granules, and not on the curdlan matrix (thermally obtained curdlan gel is a good binder for HAp granules but it does not possess any adhesive motifs, which are necessary for cell attachment, proliferation, and differentiation) [23]. One of the promising approaches to improve the cytocompatibility of polysaccharide-based biomaterials is their enrichment with peptides or proteins (e.g., collagen, gelatin, fibronectin), which contain pro-adhesive sequences. However, most peptides and proteins are thermally sensitive, which limits their use as components of scaffolds fabricated at temperatures higher than 40 °C [29]. In turn, WPI exhibits the ability to form highly cytocompatible gels during incubation of its aqueous solution at approx. 90 °C [24,25,26,27,28]. Hence, we hypothesize that addition of WPI to curdlan/HAp biomaterial enhances its cytocompatibility and biomimetic properties, as bone consists of the mineral phase (HAp), the organic phase (mainly collagen type I and noncollagenous proteins), lipids as well as water [30,31]. We also assume that the improvement of the aforementioned properties will increase with increasing content of WPI in the scaffolds. In order to verify these assumptions, the curdlan/whey protein isolate/hydroxyapatite biomaterials were evaluated by determination of their porosity, mechanical properties, water uptake ability, enzymatic degradability, protein release ability as well as cytocompatibility in vitro (evaluation of viability, proliferation, and differentiation of osteoblasts).

## 2. Materials and Methods

### 2.1. Preparation of Biomaterials

The biomaterials were fabricated with reference to guidelines described in Polish Patent no. 240,725 “Biomaterial based on natural polysaccharide—β-1,3-glucan (curdlan) and ceramics for applications in bone tissue engineering and the method of its fabrication”. Firstly, solutions of 25 wt.% and 35 wt.% WPI (BiPRO, Davisco Food International, Agropur Cooperative, Eden Prairie, MN, USA) in deionized water were obtained and subsequently they were mixed with curdlan powder (molecular weight = 80 kDa, WAKO pure Chemicals Industries, Osaka, Japan). The final concentration of curdlan in WPI solutions was 8 wt.%. Next, 1.6 g HAp granules (0.3–0.6 mm in diameter) were suspended in the obtained polymer solutions (HAp granules were fabricated by the first author according to a procedure developed previously [21]). In order to cross-link, these very dense polymer-ceramic pastes were incubated at 90 °C for 15 min (Fixed Dry Block Heater BTD Grant Instruments, Beaver Falls, PA, USA). The obtained scaffolds were cut and air-dried for 24 h. The samples were sterilized by ethylene oxide (EtO) at 55 °C for 3 h. The biomaterials were denoted as Cur/WPI_25/HAp (containing 8 wt.% of curdlan, 25 wt.% of WPI, and 1.6 g HAp) and Cur/WPI_35/HAp (containing 8 wt.% of curdlan, 35 wt.% of WPI, and 1.6 g HAp). For evaluation of structural and mechanical properties, the ability to take up liquid, and enzymatic degradation, cylinder-shaped biomaterial specimens 8 mm in diameter and 8 mm in length were used. In turn, cylinder-shaped biomaterials 8 mm in diameter and 2 mm in length were applied for assessment of their ability to release protein, bioactivity, and cytocompatibility in vitro.

### 2.2. Stuctural Characterization

The microstructure of biomaterials was evaluated using microcomputed tomography (Skyscan 1174, Bruker microCT, Kontich, Belgium). Before the experiment, the scaffolds were soaked in normal saline solution (0.9% NaCl, Sigma-Aldrich, Warsaw, Poland). The porosity (closed, open, and total) was assessed using two-dimensional (2D) image analysis. In turn, the pore size distribution was evaluated based on three-dimensional (3D) analysis, using the structure separation parameter. This parameter is defined as the diameter of the largest sphere, which fulfils two conditions: the sphere encloses the point (but the point is not necessarily the center of the sphere) and the boundary of the sphere is entirely within the pore space [32].

### 2.3. Evaluation of Mechanical Properties

The mechanical properties of biomaterials was evaluated using an Autograph AG-X Plus testing machine (Shimadzu, Kioto, Japan). Before the experiment, the scaffolds were soaked in normal saline solution (0.9% NaCl, Sigma-Aldrich, Warsaw, Poland). The measurements were performed with a load rate equal to 0.5 mm/min. The compressive strength was calculated at a strain equal to 20%. The obtained results were also used to calculate values of Young’s modulus (E).

### 2.4. Evaluation of Liquid Uptake Ability

This experiment was performed according to a procedure described in our previous work [21]. Briefly, dried biomaterials were weighed and then soaked in 0.9% NaCl solution (Sigma-Aldrich, Warsaw, Poland). After determined time points, the biomaterials were removed from the solution, slightly drained with a paper towel, and weighed. The experiment was conducted up to complete swelling of the biomaterials. The liquid uptake ability was expressed as percentage of weight increase in biomaterials over time (Wi (%)). The Wi value was calculated as described previously [21].

### 2.5. Evaluation of Ability to Release Protein

This experiment was performed in order to determine biomaterial’s ability to release protein in contact with cell culture medium. The extracts from biomaterials were prepared according to ISO 10993-12:2012 standard: Biological evaluation of medical devices–Part 12: Sample preparation and reference materials [33]. Therefore, biomaterial samples were immersed in serum-free culture media (DMEM/Ham F12 medium, Sigma-Aldrich, Warsaw, Poland or MEM alpha, Gibco, ThermoFisher Scientific, Waltham, MA, USA) in proportions equal to 0.1 g of biomaterials per 1 mL of media. The biomaterials were incubated at 37 °C for 24 h. Afterwards, the concentration of released protein (all released protein was WPI) was assessed using the Bio-Rad protein assay (Bio-Rad, Warsaw, Poland), which is an easy to use, colorimetric test based on the Bradford method. A standard curve was prepared for known concentrations of WPI solutions. According to the manufacturer’s recommendation the linearity should be achieved at protein concentrations in the range 0.05–0.5 mg/mL—thus, the standard curve of WPI was prepared in this concentration range.

### 2.6. Evaluation of Ability to Degrade Enzymatically In Vitro

The ability of biomaterials to undergo enzymatic degradation was assessed in accordance with a procedure described previously [34]. Nevertheless, for this experiment, solutions of collagenase I (320 μg/mL) or proteinase K (0.125 μg/mL) were used (both enzymes were supplied by Worthington Biochemical Corporation, Lakewood, NJ, USA). Subsequently, the biomaterials were immersed in 5 mL of enzyme solutions or PBS (control experiment), and incubated for 3, 6, and 9 weeks at 37 °C with constant agitation—50 rpm (New Brunswick^TM^ Innova^®^ 42 Incubator Shaker, Eppendorf, Warsaw, Poland). Every three weeks, the solutions of enzymes or PBS were replaced with new portions. After incubation time, the biomaterials were rinsed with PBS, air-dried, and weighed. The ability of biomaterials to degrade was assessed by the measurement of differences in the biomaterial’s weight before and after incubation in the tested solutions as described previously [34].

### 2.7. Evaluation of Bioactivity In Vitro

This experiment was carried out according to ISO 23317:2007 standard: Implants for surgery—in vitro evaluation for apatite-forming ability of implant materials [35], as described in detail previously [23]. Briefly, the simulated body fluid (SBF) was prepared based on a recipe involved in ISO 23317:2007 standard [35]. Then, biomaterials were immersed in SBF and incubated for 7, 14, and 28 days at 37 °C. The bioactivity in vitro of biomaterials was determined as the ability to form apatite layer on their polymer matrix. For this reason, the biomaterial samples were subjected to evaluation of their morphology using scanning electron microscopy (Nova NanoSEM 450, FEI) equipped with a Octane Pro EDS detector (EDAX), which enabled also chemical analysis and identification of the occurring precipitates. In other words, the data obtained from EDS allowed calculation of the Ca/P atomic ratio in order to confirm the presence of apatite layer.

### 2.8. Evaluation of Cytocompatibility In Vitro

The cell culture experiments were performed using two osteoblast cell lines, namely normal human foetal osteoblasts (hFOB 1.19, CRL-11372, ATCC, Manassas, VA, USA) and normal mouse calvarial preosteoblasts (MC3T3-E1 Subclone 4, CRL-2593, ATCC, Manassas, VA, USA). For all experiments, cells after passage 4th were used. The cells were cultured in accordance with the recommendations of the manufacturer (ATCC, Manassas, VA, USA). Therefore, hFOB 1.19 cells were grown in DMEM/Ham’s F12 medium (Sigma Aldrich, Warsaw, Poland) enriched with 300 μg/mL G418 (Sigma Aldrich, Warsaw, Poland), 10% fetal bovine serum (FBS, Pan-Biotech, Aidenbach, Germany), and antibiotics (100 U/mL penicillin and 100 μg/mL streptomycin, Sigma Aldrich, Warsaw, Poland), while MC3T3-E1 cells were cultured in MEM Alpha medium (Gibco, ThermoFisher Scientific, Waltham, MA, USA) supplemented with 10% FBS and antibiotics (100 U/mL penicillin and 100 μg/mL streptomycin). According to the manufacturer’s recommendations, the preferred conditions for cell growth were as follows: a humidified atmosphere with 5% CO_2_ at 34 °C (hFOB 1.19) or 37 °C (MC3T3-E1). The aforementioned conditions were applied for evaluation of osteoblast viability and proliferation.

In turn, for evaluation of osteoblast differentiation, hFOB 1.19 cells were used. The cells were grown in osteogenic medium, which was composed of complete culture medium (as described above) with the addition of differentiation supplements, i.e., 0.05 mg/mL ascorbic acid (Sigma-Aldrich, Warsaw, Poland), 10^−7^ M dexamethasone, and 10 mM β-glycerophosphate (Sigma-Aldrich, Warsaw, Poland). The cells were cultured in a humidified atmosphere with 5% CO_2_ at 37 °C, because ATCC indicates that hFOB 1.19 cells cultured at 37 °C proliferate slower than at 34 °C and begin the differentiation process.

#### 2.8.1. Assessment of Osteoblast Viability

The osteoblast viability was assessed via indirect contact with biomaterials (a so-called test on the liquid extracts). This assay was performed according to ISO 10093-5:2009: Biological evaluation of medical devices—Part 5: Tests for in vitro cytotoxicity [36]. The liquid extracts were prepared according to ISO 10993-12:2012 standard: Biological evaluation of medical devices—Part 12: Sample preparation and reference materials [33]. Briefly, hFOB 1.19 and MC3T3-E1 cells were seeded on 96-well plates at a concentration of 2 × 10^4^ cells/well and 3 × 10^4^ cells/well, respectively. At the same time, the biomaterials were subjected to incubation in an extraction medium −0.1 g of biomaterials were placed in 1 mL of suitable complete culture medium (culture media without biomaterials served as controls). Next, plates with cells as well as biomaterials were placed in an incubator for 24 h at 37 °C. The next day, the liquid extracts from biomaterials were collected and then culture media from cells were gently replaced with these extracts. The cells were incubated with extracts for 24 and 48 h at 34 °C (hFOB 1.19 cells) or 37 °C (MC3T3-E1 cells). After that, the cell viability was assessed by the MTT assay (Sigma-Aldrich, Warsaw, Poland), according to the procedure described previously [37].

#### 2.8.2. Assessment of Osteoblast Proliferation

The osteoblast proliferation was studied via evaluation of divisions of osteoblasts (hFOB 1.19 and MC3T3-E1) in direct contact with tested biomaterials. Therefore, hFOB 1.19 cells and MC3T3-E1 cells were seeded directly on the biomaterial surfaces at concentrations of 1 × 10^5^ cells/sample and 2 × 10^5^ cells/sample, respectively. The cells were incubated for 4 and 7 days at 34 °C (hFOB 1.19 cells) or 37 °C (MC3T3-E1 cells). The cell proliferation was estimated using a Cell counting Kit-8 (WST-8, Sigma-Aldrich, Warsaw, Poland). Based on the obtained optical density (OD) values, the fold increase in cell proliferation (FI) was determined, as described previously [22].

Additionally, in order to visualize the osteoblast morphology, cell nuclei and cytoskeletons (F-actin filaments) were stained with Hoechst 33342 (Sigma-Aldrich, Warsaw, Poland) and AlexaFluor^TM^ 635 Phalloidin dyes, respectively. The cells were observed under a confocal laser scanning microscope (CLSM, Olympus Fluoview equipped with FV1000, Shinjuku, Japan).

#### 2.8.3. Assessment of Osteoblast Differentiation

The osteogenic differentiation was assessed quantitatively (ELISA tests) and qualitatively (CLSM observations) using human osteoblasts. The hFOB 1.19 cells were seeded directly on the biomaterial surfaces at a concentration of 1 × 10^5^ cells/sample and cultured for 7, 14, and 21 days at 37 °C. The osteogenic medium was changed every third day of the experiment. The ELISA tests (ELISA Kit for Collagen Type I—COL1, ELISA Kit for Bone Alkaline Phosphatase—bALP, and ELISA Kit for Osteocalcin—OC, Cloud-Clone Corp., Wuhan, Hubei) were performed using cell lysates, which were prepared according to the protocol described in detail by Przekora and Ginalska [38]. Moreover, after 21-day incubation, osteogenic markers were visualized using immunofluorescence staining according to procedure described previously [34]. In this case, rabbit polyclonal anti-collagen I antibody (Invitrogen, ThermoFisher Scientific, Waltham, MA, USA), rabbit polyclonal anti-osteocalcin antibody (Bioss, ThermoFisher Scientific, Waltham, MA, USA), and goat anti-rabbit IgG (H + L) antibody-conjugated with AlexaFluor^®^ 488 (Abcam, Cambridge, UK) were used. Cell nuclei were dyed with Hoechst 33342 (Sigma-Aldrich, Warsaw, Poland). The cells were observed under CLSM (Olympus Fluoview equipped with FV1000, Shinjuku, Japan).

### 2.9. Statistical Analysis

The analysis was conducted in at least three independent experiments and obtained results were expressed as mean values ± standard deviation (SD). The normal distribution of data was analyzed using a D’Agostino and Pearson omnibus normality test. The statistical analysis was carried out using unpaired Student’s *t*-test or One-Way ANOVA test, followed by a Tukey’s multiple comparison test. The differences between investigated groups were recognized as statistically significant when *p* < 0.05 (GraphPad Prism 5, Version 5.04, GraphPad Software, San Diego, CA, USA).

## 3. Results and Discussion

### 3.1. Pore Size Distribution and Porosity

**Figure 1:**
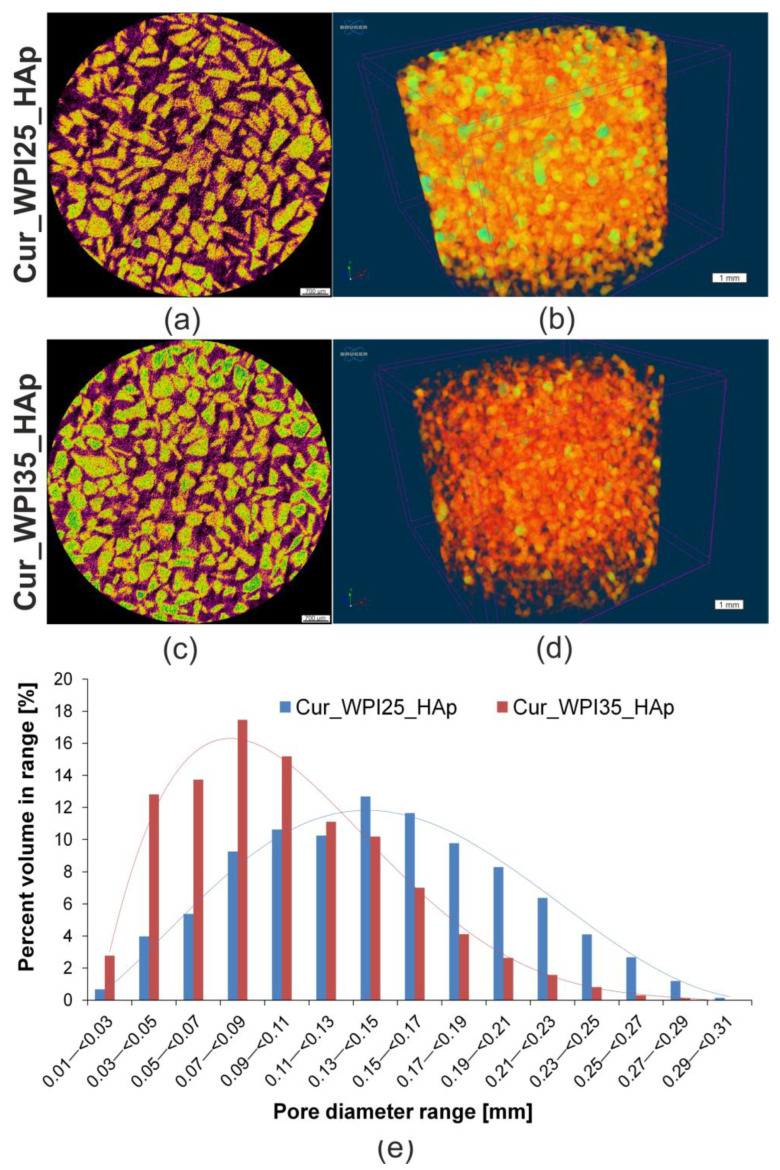
Microcomputed tomography (microCT) analysis of Cur_WPI25_HAp and Cur_WPI35_HAp scaffolds. (**a**,**b**) Cross-sections of biomaterials (scale bar = 700 μm), yellow color—HAp granules, purple color—curdlan/WPI matrix, black color—air voids; (**c**,**d**) 3D architecture of biomaterials (scale bar = 1 mm); (**e**) The distribution of pore sizes within biomaterials.

The two-dimensional (2D) microcomputed tomography (microCT) analysis showed that HAp granules (visible as yellow color) were evenly distributed within the curdlan/WPI matrixes (visible as purple color) of both biomaterials (Figure 1a,c). In turn, the three-dimensional (3D) microCT analysis (Figure 1b,d) allowed determination of the pore size distribution within biomaterials (Figure 1e) and their porosity. It was demonstrated that the Cur_WPI25_HAp biomaterial possessed pores with a higher size (average range equal to approx. 130–150 μm) compared to the Cur_WPI35_HAp biomaterial (average range equal to approx. 70–100 μm). Moreover, it was found that the total porosity of the Cur_WPI25_HAp biomaterial (approx. 37%) was greater than the porosity of the Cur_WPI35_HAp scaffold (approx. 25%) (Table 1). The porosity and pore size are very important structural features, which scaffolds intended for bone regeneration should have, as bone is a porous tissue. Cortical bone possesses a lower porosity (5–10%) and smaller pore sizes (10–50 μm) compared to cancellous bone (porosity close to 75–90%, pore sizes equal to approx. 300–600 μm) [39]. Thus, it is considered that the presence of pores with sizes ranging from 100 to 350 μm influences mainly the osteoconductive and osteoinductive properties of biomaterials, because such pores allow cell migration through the structure of scaffolds, support proliferation, and differentiation of cells as well as promoting production of ECM. In turn, the pores with sizes above 300 μm enable ingrowth of new bone and formation of new blood vessels [40,41]. Therefore, the bone scaffolds should be porous and should be characterized by the presence of pores that have sizes at least equal to 100 μm [42]. Based on data obtained for Cur_WPI25_HAp and Cur_WPI35_HAp biomaterials, it can be concluded that they meet minimal pore size requirements for bone scaffolds (average pore sizes about 100 μm), but they could be more porous (ideally, the porosity of biomaterials should be close to that of cancellous bone). In future, we plan to improve the porosity of our biomaterials by using different, more advanced fabrication procedures including *inter alia* electrospinning and 3D printing techniques [43].

### 3.2. Young’s Modulus and Compressive Strength

**Figure 2:**
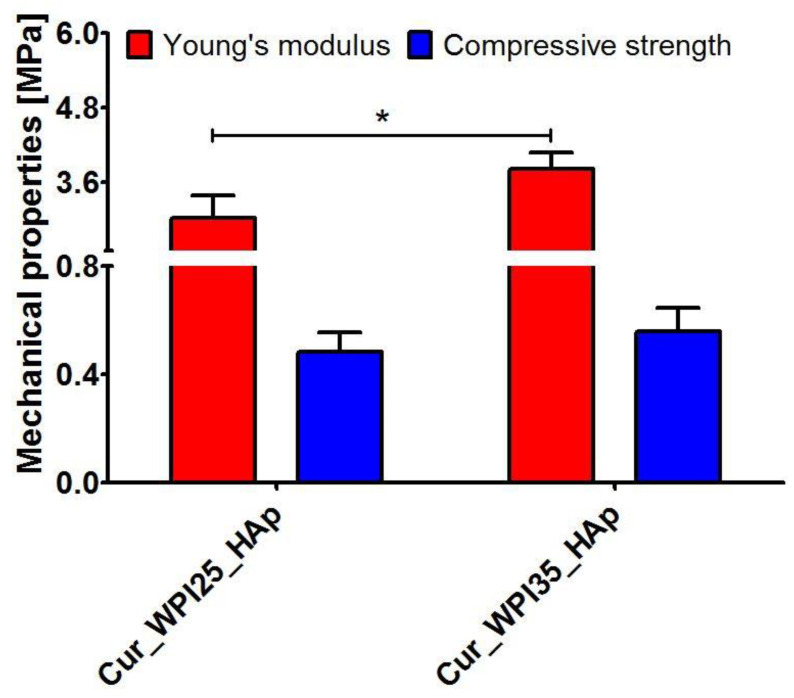
Young’s modulus and compressive strength of Cur_WPI25_HAp and Cur_WPI35_HAp biomaterials. The unpaired Student’s *t*-test was performed in order to determine statistical differences between samples, *p* < 0.05: * Significantly different results between values of Young’s modulus of Cur_WPI25_HAp and Cur_WPI35_HAp biomaterials.

The mechanical tests (Figure 2) indicated that the Young’s modulus of the Cur_WPI35_HAp biomaterial (3.81 ± 0.26 MPa) was significantly greater (*p* < 0.05) compared to that of the Cur_WPI25_HAp scaffold (3.02 ± 0.37 MPa). Moreover, the Cur_WPI35_HAp biomaterial was characterized by higher compressive strength (0.56 ± 0.09 MPa) compared to the Cur_WPI25_HAp scaffold (0.48 ± 0.07 MPa), but the differences were not statistically significant (*p* > 0.05). It must be noted that the fabricated biomaterials possessed significantly lower mechanical properties compared to natural bone as Young’s modulus and compressive strength for cortical bone are 18–22 GPa and 110–150 MPa and for cancellous bone are 0.1–0.3 GPa and 2–6 MPa, respectively [44]. Nevertheless, their mechanical properties were better than those of other bone biomaterials composed of curdlan and hydroxyapatite granules. In our previous study, we demonstrated that Young’s modulus and compressive strength of curdlan/HAp biomaterial obtained via dialysis against calcium chloride solution were 0.17 ± 0.05 MPa and 0.057 ± 0.01 MPa, respectively [21]. In turn, Borkowski et al. showed that Young’s modulus and compressive strength values of a thermally obtained biomaterial composed of curdlan and HAp granules were close to 0.55 MPa and 0.25 MPa, respectively [45]. Hence, these results proved that addition of WPI to curdlan-based biomaterials improved their mechanical properties, namely the higher concentration of WPI led to better mechanical properties of the curdlan-based biomaterial. It is an important feature of WPI, because in general, addition of proteins such as collagen or gelatin to polymer-based biomaterials decreases their mechanical properties [29]. Nevertheless, despite the fact that curdlan-based biomaterials enriched with WPI possessed better values of Young’s modulus and compressive strength compared to the aforementioned curdlan-based scaffolds, their mechanical properties were still lower in comparison with natural bone. Therefore, their application should focus on non-load bearing implantation sites. For instance, they may be used as scaffolds for craniofacial bone tissue engineering applications [21], but their use should be supported by additional application of mechanically strong titanium plates [46].

### 3.3. Swelling Ability

**Figure 3:**
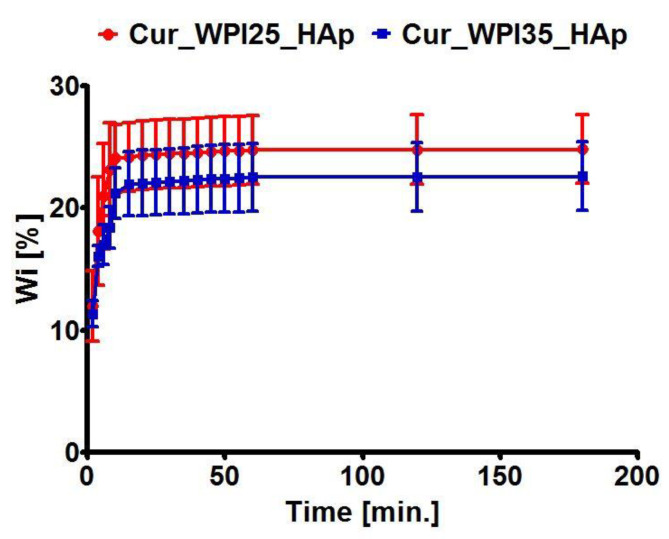
Swelling ability of Cur_WPI25_HAp and Cur_WPI35_HAp biomaterials. The scaffolds were immersed in 0.9% NaCl solution and weighed at specific time points. No significant differences between biomaterials were obtained.

The liquid uptake test showed that both biomaterials possessed the ability to swell (Figure 3), whereby the Cur_WPI25_HAp biomaterial possessed only a slightly better ability to absorb 0.9% NaCl solution (*p* > 0.05) in comparison with the Cur_WPI35_HAp scaffold. In general, their weight increased by approx. 15% compared to initial masses and complete swelling was reached within 15 min. The evaluation of the swelling ability of biomaterials is very important because it allows estimation of the time needed for biomaterial immersion in, e.g., NaCl solution, antibiotic solution or blood before implantation. It is assumed that the biomaterial should achieve complete saturation in no more than 30 min before implantation [47]. Considering the above, both scaffolds can be considered as potential implantable biomaterials.

### 3.4. Capacity to Release Protein

**Figure 4:**
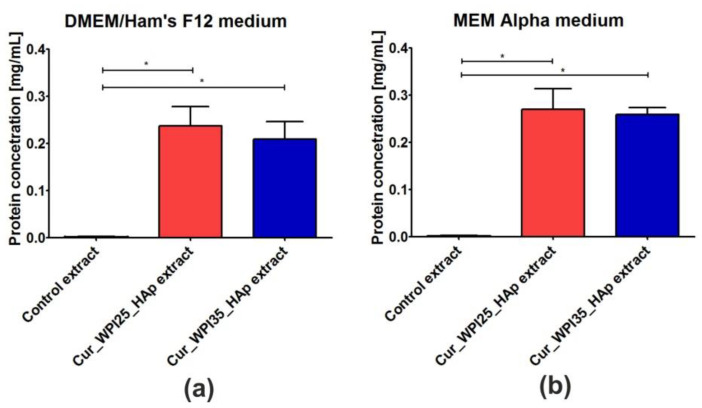
The ability of Cur_WPI25_HAp and Cur_WPI35_HAp biomaterials to release protein in a liquid environment. The extracts from biomaterials were prepared according to ISO 10993-12:2012 standard: Biological evaluation of medical devices—Part 12: Sample preparation and reference materials [33]. The biomaterial samples were immersed in serum-free culture media intended for osteoblast culture (DMEM/Ham F12 medium (**a**) or MEM alpha medium (**b**)) in the proportion equal to 0.1 g of biomaterials per 1 mL of media. Control extracts were obtained by incubation of culture media in non-cytotoxic polystyrene cell culture plate. The biomaterials were incubated at 37 °C for 24 h. The unpaired Student’s *t*-test was performed in order to determine statistical differences between samples, *p* < 0.05: * Significantly different results between concentration of protein in extracts obtained from Cur_WPI25_HAp and Cur_WPI35_HAp biomaterials and protein content in culture media.

The ability of biomaterials to release protein (WPI) was assessed using liquid extracts prepared in the cell culture media, namely DMEM/Ham’s F12 medium intended for culture of hFOB 1.19 osteoblasts (Figure 4a) and MEM Alpha medium recommended for culture of MC3T3-E1 osteoblasts (Figure 4b). It was demonstrated that both biomaterials exhibited the ability to release significant amounts of protein into culture media (*p* < 0.05). Although the Cur_WPI25_HAp biomaterial secreted a slightly greater amount of WPI compared to the Cur_WPI35_HAp scaffold, the differences between samples were insignificant (*p* > 0.05). This experiment was performed in order to determine whether addition of WPI to curdlan-based scaffold had only a direct effect or also an indirect influence on cell responses. Because the biomaterials had the ability to release significant amounts of WPI into cell culture media, it is expected that such extracts may have a beneficial influence on cell viability (this will be evaluated in Section 3.7. Viability of osteoblasts).

### 3.5. Biodegradation In Vitro

**Figure 5:**
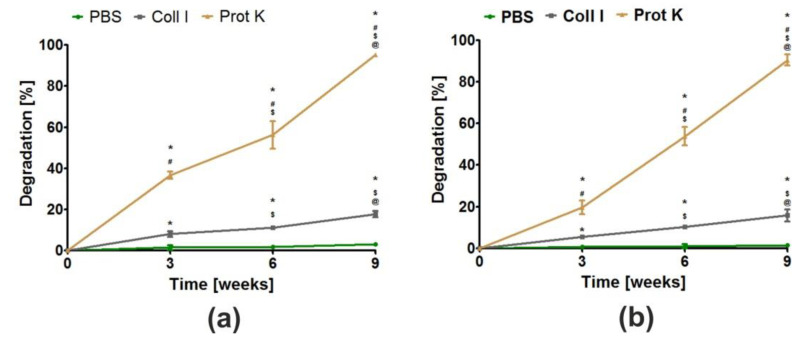
The ability of Cur_WPI25_HAp (**a**) and Cur_WPI35_HAp (**b**) biomaterials to degrade in PBS (control), collagenase I (Coll I), and proteinase K (Prot K) solutions during 3, 6, and 9 weeks of incubation. The normal distribution of data was analyzed using a D’Agostino and Pearson omnibus normality test, then one-way ANOVA test followed by Tukey’s multiple comparison were performed in order to determine statistical differences between samples, *p* < 0.05: * Significantly different results compared to results obtained in PBS solution; ^#^ significantly different results compared to results obtained in collagenase I solution; ^$^ significantly different results between data obtained after 3 weeks; ^@^ significantly different results between data obtained after 6 weeks.

Biodegradation is the ability of biomaterials to break down in a controlled manner under in vivo conditions, in order to create space for new bone tissue. The rate of scaffold degradability should be compatible with the proliferation of osteoblasts, as the cells need empty spaces for division and formation of ECM [13,15,48]. After implantation, the scaffolds may be degraded via chemical and enzymatic oxidation, nonenzymatic hydrolysis as well as enzyme-catalyzed hydrolysis. Enzymatic hydrolysis is mainly catalyzed by proteases, esterases, glycosidases or phosphatases [49]. For evaluation of the potential ability of biomaterials to degrade, this in vitro experiment was performed using two solutions of proteases (collagenase I and proteinase K). Both collagenase I and proteinase K solutions are successfully used for assessment of degradability in vitro of bone scaffolds [50,51]. Firstly, it was demonstrated that both biomaterials were stable in control solution (PBS) during 9 weeks of the experiment (Figure 5a,b). Secondly, the obtained results showed that both biomaterials possessed the ability to undergo enzymatic degradation in vitro, whereby the weight loss of the Cur_WPI25_HAp biomaterial (Figure 5a) and the Cur_WPI35_HAp (Figure 5b) scaffold in collagenase I solution was significantly lower compared to the weight loss of these biomaterials after incubation in proteinase K solution. This phenomenon was expected, as proteinase K possesses a higher enzymatic activity compared to collagenase I [52]. After 9 weeks of incubation of biomaterials in collagenase I solution, the degradation of Cur_WPI25_HAp and Cur_WPI35_HAp biomaterials was close to 18% and 15%, respectively. In turn, incubation of biomaterials in proteinase K solution for 3 and 6 weeks led to losses of the biomaterial’s weight equal to approx. 36% and 56% (Cur_WPI25_HAp biomaterial) and 21% and 55% (Cur_WPI35_HAp biomaterial). At the end of the experiment (after 9 weeks), the biomaterials soaked in proteinase K solution were almost completely broken down (only a small amount of biomaterial residuals were present). Moreover, it was noted that the degradation rate of the Cur_WPI25_HAp biomaterial in both enzyme solutions was slightly faster compared to the degradation rate of the Cur_WPI35_HAp scaffold. This fact is probably associated with the slightly greater porosity and swelling ability of the Cur_WPI25_HAp biomaterial compared to the Cur_WPI35_HAp scaffold (Table 1, Figure 3). Similar observations were reported by Kang et al. [53], who evaluated degradability of gelatin-based biomaterials. The authors indicated that the degradation rate of biomaterials decreased with the increase in gelatin concentration in scaffolds, which was associated with their lower porosity and swelling ability. To sum up, this experiment proved that both investigated biomaterials have the ability to undergo enzymatic degradation in vitro in a controllable manner, as the loss of their masses decreased gradually over the duration of the experiment.

### 3.6. Apatite Formation Ability

**Figure 6:**
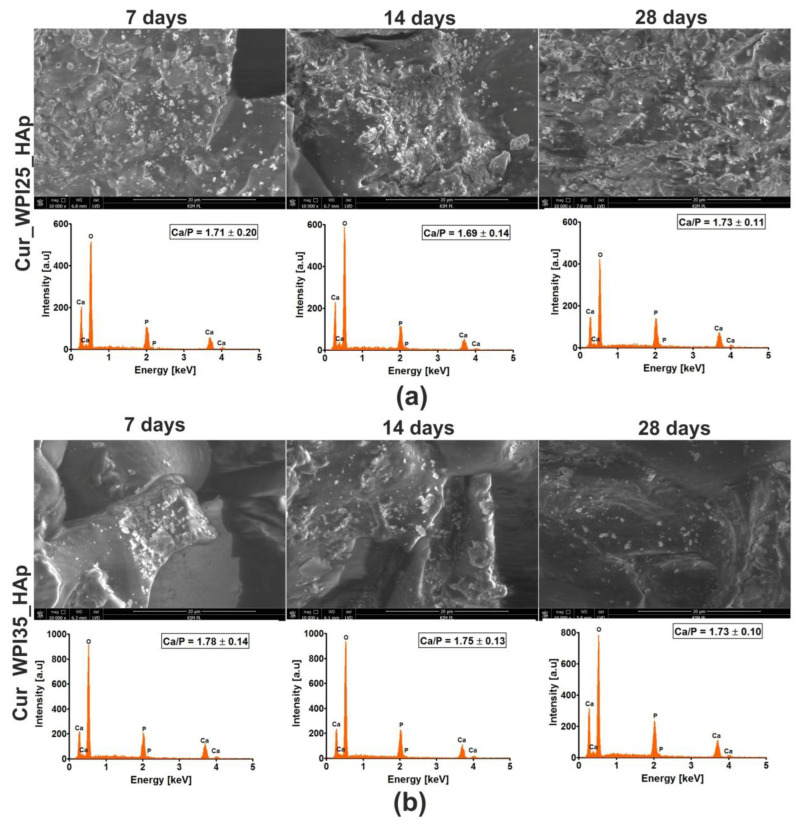
Scanning electron microscopy (SEM) images and Energy Dispersive Spectroscopy (EDS) spectra of Cur_WPI25_HAp (**a**) and Cur_WPI35_HAp (**b**) biomaterials after incubation in simulated body fluid (SBF). The experiment was performed for 7, 14, and 28 days according to ISO 23317:2007 standard: Implants for surgery—In vitro evaluation for apatite-forming ability of implant materials [35]. Magnification of SEM images = 10,000×; scale bar = 20 μm.

The bioactivity of a biomaterial in vitro is defined as its ability to form an apatite layer on its surface. Thanks to the presence of apatite, strong, multi-phase bonds between scaffold and surrounding bone tissue may be created. The formation of this tight connection between biomaterial and bone (also called osseointegration) is necessary for proper healing and regeneration of bone tissue in vivo [17,18]. This apatite formation ability experiment in vitro indicated that both biomaterials possessed the ability to create apatite on their curdlan-WPI matrixes even after 7 days of incubation (Figure 6a,b). EDS measurements demonstrated that visible crystals were composed of calcium and phosphorus with similar Ca/P ratios ranging from 1.69–1.78. These ratios were very similar to the Ca/P ratio determined for natural hydroxyapatite (1.67) [23]. Hence, EDS results suggest the formation of a calcium phosphate phase. If this is apatite, it is highly possible that after implantation, these biomaterials will form an apatite layer on their surfaces and then they will be osseointegrated into surrounding bone tissue. Interestingly, in our previous research, we demonstrated that thermally obtained scaffolds composed of curdlan and HAp granules did not possess the ability to form apatite. Indeed, no presence of apatite was observed even after 28 days of incubation in SBF solution [23]. Thus, the results obtained in this research indicate that the addition of WPI to a curdlan matrix enhances its bioactivity.

### 3.7. Viability of Osteoblasts

**Figure 7:**
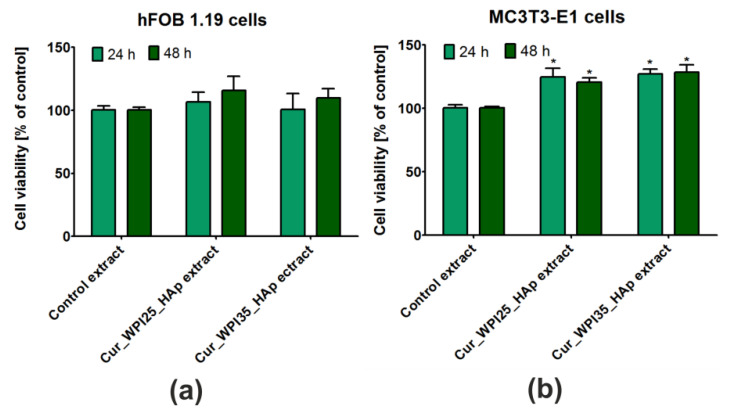
Viability of (**a**) normal human foetal osteoblasts (hFOB 1.19, ATCC CRL-11372) and (**b**) normal mouse calvarial preosteoblasts (MC3T3-E1 Subclone 4, ATCC CRL-2593) after 24 and 48 h incubation with extracts obtained from Cur_WPI25_HAp and Cur_WPI35_HAp biomaterials. The extracts from biomaterials were prepared according to ISO 10993-12:2012 standard: Biological evaluation of medical devices—Part 12: Sample preparation and reference materials [33]. Control extracts were obtained by incubation of culture media in non-cytotoxic polystyrene culture plate. The biomaterials were incubated at 37 °C for 24 h. Cell viability was assessed using the MTT assay. The unpaired Student’s *t*-test was performed in order to determine statistical differences between samples, *p* < 0.05: * Significantly different results compared to control extracts.

The MTT assay was performed after treatment of cells with liquid extracts from biomaterials in order to determine whether the investigated scaffolds may have an indirect influence on osteoblast viability (Figure 7a,b). In the case of human osteoblasts (hFOB 1.19 cells), it was shown that extracts obtained from the investigated biomaterials promoted their viability (Figure 7a), but the differences were not statistically significant (*p* > 0.05) compared to the effect obtained for the control extract (culture medium incubated in polystyrene culture plate). In turn, these extracts exerted a significant influence (*p* < 0.05) on the viability of mouse preosteoblasts (MC3T3-E1 cells) in comparison with control extracts (Figure 7b). Hence, the viability of mouse osteoblasts after 24 and 48 h of incubation with extracts was as follows: 100.50 ± 3.91% and 100.40 ± 2.02% (exposure to control extract); 127.70 ± 11.95% and 120.50 ± 6.42% (exposure to extracts from the Cur_WPI25_HAp biomaterial); 127.10 ± 6.87% and 128.40 ± 10.09% (exposure to the Cur_WPI35_HAp biomaterial). Hence, the obtained results confirmed our hypothesis posed in the previous experiment (Section 3.4. Capacity to release protein), namely, we proved that WPI released from biomaterials to culture medium stimulates osteoblast viability.

### 3.8. Proliferation of Osteoblasts

**Figure 8:**
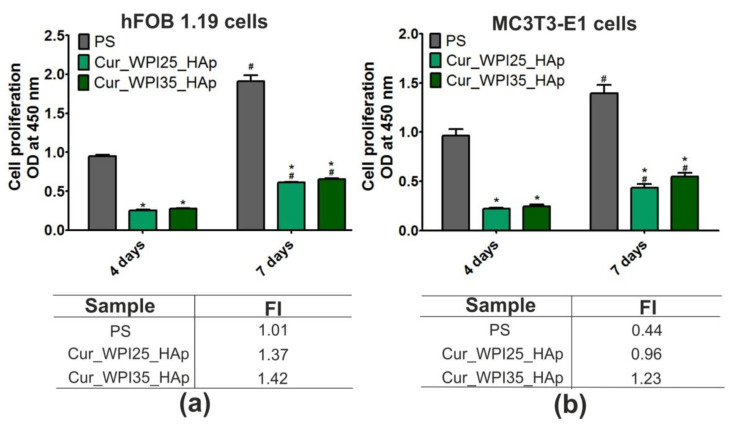
Proliferation of (**a**) normal human foetal osteoblasts (hFOB 1.19, ATCC CRL-11372) and (**b**) normal mouse calvarial preosteoblasts (MC3T3-E1 Subclone 4, ATCC CRL-2593) after 4 and 7 days of cell culture on polystyrene (PS, control), Cur_WPI25_HAp, and Cur_WPI35_HAp biomaterials. Cell proliferation was assessed using the WST-8 assay. The normal distribution of data was analyzed using a D’Agostino and Pearson omnibus normality test, then one-way ANOVA test followed by Tukey’s multiple comparison were performed in order to determine statistical differences between samples, *p* < 0.05: * Significantly different results between Cur_WPI25_HAp or Cur_WPI35_HAp biomaterials and polystyrene (PS) at the same time of experiment; ^#^ significantly different results between results obtained after 4 and 7 days of incubation. FI denoted fold increase in cell proliferation.

The colorimetric assay (i.e., WST-8 test) indicated that both Cur_WPI25_HAp and Cur_WPI35_HAp biomaterials promoted proliferation of hFOB 1.19 cells (Figure 8a) and MC3T3-E1 cells (Figure 8b), as the metabolic activities of cells (proportional to OD values) after 7 days of culture were significantly higher (*p* < 0.05) compared to metabolic activities of cells after 4 days of incubation. Although the metabolic activities of both hFOB 1.19 cells and MC3T3-E1 cells grown on the Cur_WPI35_HAp biomaterial were greater compared to the metabolic activities of these cells cultured on the Cur_WPI25_HAp scaffold, no significant differences (*p* > 0.05) were noted. At the end of the experiment, the OD values for hFOB 1.19 cells were approx. 0.613 (cells on the Cur_WPI25_HAp biomaterial) and 0.654 (cells on the Cur_WPI35_HAp scaffold), while the OD values for MC3T3-E1 cells were approx. 0.435 (cells on the Cur_WPI25_HAp biomaterial) and approx. 0.549 (cells on the Cur_WPI35_HAp scaffold), respectively. Importantly, the calculated values of FI (i.e., fold increase in cell proliferation) indicated that the division rates of both types of osteoblasts cultured on Cur_WPI25_HAp and Cur_WPI35_HAp biomaterials were greater compared to the division rates of these cells seeded on polystyrene (PS). Among the tested biomaterials, the Cur_WPI35_HAp scaffold allowed faster osteoblast divisions compared to the Cur_WPI_25_HAp biomaterial.

**Figure 9:**
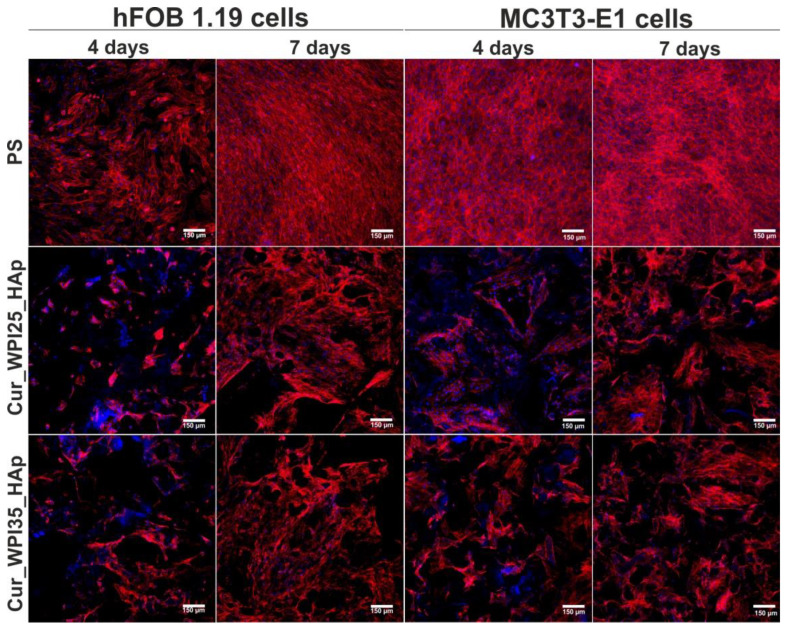
Confocal microscopy images demonstrating morphology of normal human foetal osteoblasts (hFOB 1.19, ATCC CRL-11372) and normal mouse calvarial preosteoblasts (MC3T3-E1 Subclone 4, ATCC CRL-2593) cultured on polystyrene (PS, control), Cur_WPI25_HAp, and Cur_WPI35_HAp biomaterials after 4 and 7 days of incubation. Nuclei gave blue fluorescence (sporadically visible, additional blue fluorescence was an effect of presence of WPI in the structure of biomaterials), while cytoskeletal filaments emitted red fluorescence; magnification 100×, scale bar = 150 μm.

Confocal microscope observations (Figure 9) were in good agreement with the results obtained during the WST-8 assay (Figure 8a,b). Firstly, it was found that both hFOB 1.19 cells and MC3T3-E1 cells grown on PS (control), Cur_WPI25_HAp, and Cur_WPI35_HAp biomaterials possessed a normal morphology—they were flattened and well-spread, which indicated that both types of osteoblasts had beneficial conditions for growth and proliferation. Moreover, it was observed that the number of cells increased with an extension of the incubation time. Most importantly, the hFOB 1.19 cells and MC3T3-E1 cells seeded on polymer-ceramic scaffolds were visible on both HAp granules and curdlan-WPI matrixes (especially at day 7), which revealed that the microstructure of the investigated biomaterials promoted cell grown. As we aforementioned, our previous research including the evaluation of cytocompatibility of thermally obtained scaffolds for bone regeneration composed only of HAp granules and curdlan (glu/HA T) showed that this biomaterial promoted proliferation of hFOB 1.19 cells and MC3T3-E1 cells, but both types of osteoblasts preferentially settled on the surface of HAp granules (unlike curdlan matrix), which indicated that the curdlan matrix was not suitable for cell growth and proliferation [23]. In turn, the current study clearly demonstrated that the combination of WPI with curdlan increased cytocompatibility of the polymer-based matrix in the polymer-ceramic biomaterials. Thus, the biomaterials investigated in this study (Cur_WPI25_HAp and Cur_WPI35_HAp scaffolds) composed of protein (WPI), polysaccharide (curdlan), and HAp granules seem to be more promising for bone tissue engineering in comparison with the biomaterial comprised only of curdlan and HAp granules.

### 3.9. Osteogenic Differentiation of Osteoblasts

**Figure 10:**
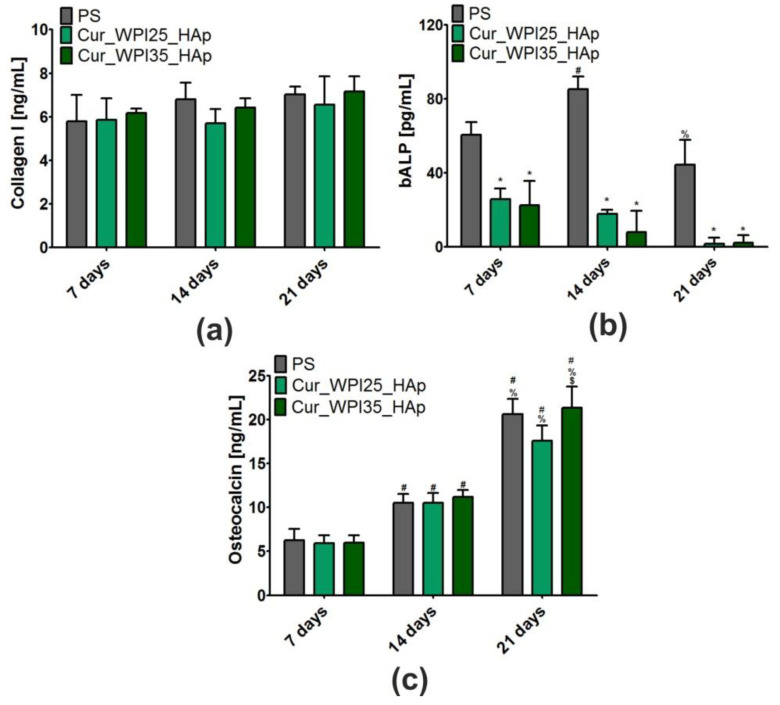
Levels of osteogenic markers: (**a**) collagen I (COLI), (**b**) bone alkaline phosphate (bALP), and (**c**) osteocalcin (OC) in normal human foetal osteoblasts (hFOB 1.19, ATCC CRL-11372) after 7, 14, and 21 days of cell culture on polystyrene (PS, control), Cur_WPI25_HAp, and Cur_WPI35_HAp biomaterials. Results were obtained using ELISA tests. The normal distribution of data was analyzed using a D’Agostino and Pearson omnibus normality test, then one-way ANOVA test followed by Tukey’s multiple comparison were performed in order to determine statistical differences between samples, *p* < 0.05: * Significantly different results between Cur_WPI25_HAp or Cur_WPI35_HAp biomaterials and polystyrene (PS) at the same time of experiment; ^$^ significantly different results between Cur_WPI25_HAp and Cur_WPI35_HAp biomaterials; ^#^ significantly different results compared to data obtained after 7 days; ^%^ significantly different results compared to data obtained after 14 days.

**Figure 11:**
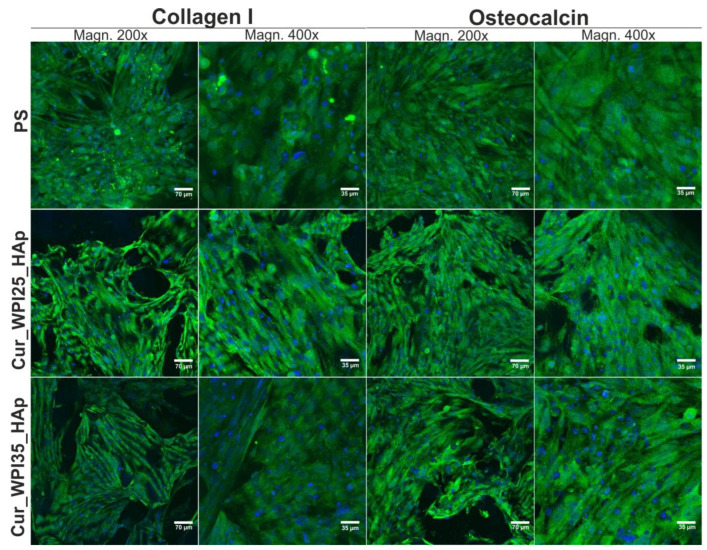
Confocal microscopy images demonstrating osteogenic markers (collagen I and osteocalcin) produced by human foetal osteoblasts (hFOB 1.19, ATCC CRL-11372) cultured on polystyrene (PS, control), Cur_WPI25_HAp, and Cur_WPI35_HAp biomaterials after 21 days incubation. Nuclei gave blue fluorescence, while collagen I or osteocalcin emitted green fluorescence; magnification 200× and 400×, scale bar = 70 μm and 35 μm.

In this experiment, levels of three typical osteogenic markers—collagen I (COLI), bone alkaline phosphate (bALP), and osteocalcin (OC) in hFOB 1.19 cells were evaluated using ELISA tests (Figure 10a–c). In the case of COLI produced by osteoblasts cultured on PS, Cur_WPI25_HAp, and Cur_WPI35_HAp biomaterials, it was demonstrated that its levels were almost the same during the whole duration of the experiment (Figure 10a). Thus, no significant differences between investigated samples or between tested time intervals were observed. In contrast, significant differences in bALP levels were noted (Figure 10b). Primarily, it was observed that despite the increasing duration of the experiment, the levels of this marker in cells cultured on both polymer-ceramic scaffolds were significantly lower (*p* < 0.05) compared to amounts of bALP secreted by osteoblasts grown on PS. Moreover, it was found that the activity of bALP in cells cultured on PS significantly increased from day 7 to 14 (from 60.56 ± 6.97 pg/mL to 85.25 ± 6.69 pg/mL, *p* < 0.05), and then declined to 44.25 ± 13.56 pg/mL (*p* < 0.05 between results obtained after 14 and 21 days incubation). In contrast, the highest bALP levels (approx. 22 pg/mL) in cells grown on both scaffolds were detected on the 7th day of experiment. Next, the amount of bALP secreted by osteoblasts cultured on biomaterials was constantly decreasing. In turn, hFOB 1.19 cells cultured on PS and investigated scaffolds exhibited similar tendency to synthesize OC (Figure 10c)—levels of this marker increased along with the extension of the duration of the experiment. On the 21st day, the amounts of OC synthesized by cells cultured on PS, Cur_WPI25_HAp, and Cur_WPI35_HAp were 20.61 ± 1.76 ng/mL, 17.58 ± 2.02 ng/mL, and 21.31 ± 2.44 ng/mL. Thus, the level of OC in cells cultured on the Cur_WPI35_HAp biomaterial was slightly higher compared to the amounts of this protein produced by osteoblasts grown on PS and the Cur_WPI25_HAp scaffold. In order to confirm the quantitative data, the immunofluorescence staining of collagen I and osteocalcin in cells was performed on day 21 (Figure 11). Hence, it was clearly shown that osteoblasts cultured on both PS (control) as well as on Cur_WPI25_HAp and Cur_WPI35_HAp scaffolds produced high amounts of the aforementioned proteins, which indicated that the results obtained from ELISA tests are in good correlation with confocal microscope observations.

It is worth underlining that osteoblast differentiation is a complex, three-stage process, which may be regulated by composition of biomaterials as well as their structural, physicochemical, and mechanical properties [21,38,54]. In brief, at the beginning of differentiation, the osteoblasts mainly synthesize collagen I. Then, during the second stage of differentiation, the cells secrete high amounts of bALP. In turn, at the end of this process, the level of bALP decreases and osteoblasts produce a great amount of proteins involved in calcification of ECM (e.g., osteocalcin) [21,38,54]. Considering all the results obtained in this study (Figure 10a–c and Figure 11), it may be stated that both PS (control) and the biomaterials allowed proper differentiation of human osteoblasts. Nevertheless, this process was slightly different in cells cultured on PS (two-dimensional culture) compared to the process in cells grown on scaffolds (three-dimensional culture). Based on data of bALP levels (Figure 10b), it seems that the second stage of differentiation in cells cultured on scaffolds began earlier compared to osteoblasts grown on PS, as the highest bALP activities were detected on 7th day and 14th day, respectively. After that, bALP levels in cells declined and at the same time the amounts of secreted osteocalcin increased (Figure 10c), which indicated that the third state of differentiation (also called ECM mineralization) had started. According to the presented results, it is difficult to determine which polymer-ceramic scaffold more potently supported osteoblast differentiation. Nevertheless, it is important to highlight that both biomaterials allowed normal differentiation of osteoblasts, which further confirmed their high cytotocompatibility in vitro.

## 4. Conclusions

In this paper, we assessed the main features of novel biomaterials composed of curdlan, WPI, and HAp granules (namely Cur_WPI25_HAp and Cur_WPI35_HAp scaffolds) in the context of their potential applications in bone tissue engineering. We determined that both biomaterials—as with most polymer-based biomaterials—were characterized by relatively low porosity and weak mechanical properties in comparison with natural bone. On the other hand, these biomaterials also possessed many desirable properties. Both scaffolds possessed the ability to absorb low amounts of liquid over an acceptable time period and to release a high amount of WPI to the surrounding aqueous environment. It was also demonstrated that the scaffolds underwent controllable degradation in collagenase I and proteinase K solutions within 9 weeks. Moreover, the bioactivity assay suggested that both biomaterials formed an apatite layer on their surfaces in vitro after just 7 days of incubation in SBF. Hence, one can suppose that after their implantation they will form an apatite on their surfaces and be integrated with surrounding bone tissue. In turn, cell culture experiments showed that both biomaterials were characterized by high cytocompatibility in vitro. The MTT assay demonstrated that even extracts obtained from biomaterials supported cell viability, while the WST-8 assay and confocal microscopy observations indicated that the surfaces of both biomaterials promoted osteoblast proliferation over time. Both biomaterials also allowed production of characteristic osteogenic markers by osteoblasts, as proven by ELISA assays and confocal microscopy observations. Considering all the obtained results, it seems that both biomaterials meet many requirements for bone scaffolds. However, their porosity and mechanical properties should be improved, which will be the subject of our further work.

## 5. Patents

The biomaterials were fabricated according to the method described in Polish Patent no. 240,725 “Biomaterial based on natural polysaccharide—β-1,3-glucan (curdlan) and ceramics for applications in bone tissue engineering and the methods of its fabrication”.

## Figures and Tables

**Table 1:** Porosity of Cur_WPI25_HAp and Cur_WPI35_HAp biomaterials.

Scaffold	Porosity [%] ± SD		
	Closed ^1^	Open ^2^	Total ^3^
Cur_WPI25_HAp	8.09 ± 4.30	29.13 ± 5.49	37.21 ± 1.59
Cur_WPI35_HAp	21.04 ± 1.80	3.95 ± 1.85	24.99 ± 1.44

^1^ Closed porosity—the porosity determined by presence of pores that are completely isolated from the external surface of the biomaterial. ^2^ Open porosity—the porosity determined by presence of pores that are connected to the external surface of the biomaterial. ^3^ Total porosity—the sum of closed and open porosity.

## Data Availability

Data are available on reasonable request. The data can be obtained from K.K.
